# Binding modes of environmental endocrine disruptors to human serum albumin: insights from STD-NMR, ITC, spectroscopic and molecular docking studies

**DOI:** 10.1038/s41598-017-11604-3

**Published:** 2017-09-11

**Authors:** Hongqin Yang, Yanmei Huang, Jiuyang Liu, Peixiao Tang, Qiaomei Sun, Xinnuo Xiong, Bin Tang, Jiawei He, Hui Li

**Affiliations:** 1 College of Chemical Engineering, Sichuan University, Chengdu, 610065 China; 2 School of Life Sciences, University of Science and Technology of China, Hefei, 230026 China

## Abstract

Given that bisphenols have an endocrine-disrupting effect on human bodies, thoroughly exposing their potential effects at the molecular level is important. Saturation transfer difference (STD) NMR-based binding studies were performed to investigate the binding potential of two bisphenol representatives, namely, bisphenol B (BPB) and bisphenol E (BPE), toward human serum albumin (HSA). The relative STD (%) suggested that BPB and BPE show similar binding modes and orientations, in which the phenolic rings were spatially close to HSA binding site. ITC analysis results showed that BPB and BPE were bound to HSA with moderately strong binding affinity through electrostatic interactions and hydrogen bonds. The order of binding affinity of HSA for two test bisphenols is as follows: BPE > BPB. The results of fluorescence competitive experiments using 5-dimethylaminonaphthalene-1-sulfonamide and dansylsarcosine as competitors, combined with molecular docking indicated that both bisphenols are prone to attach to the binding site II in HSA. Spectroscopic results (FT-IR, CD, synchronous and 3D fluorescence spectra) showed that BPB/BPE induces different degrees of microenvironmental and conformational changes to HSA.

## Introduction

Endocrine disruptors (EDs) are exogenous substances that interfere with hormone biosynthesis and metabolism or cause deviation from normal homeostatic control or reproduction^1, 2^. Animal models, human clinical observations, and epidemiological studies implicate EDs as a significant public health concern^3^. EDs include hundreds of chemicals, estrogen-mimicking compounds, and xenoestrogens; bisphenol A (BPA) is a prototype of xenoestrogenic ED^4, 5^. Bisphenol A is an industrially important chemical that is abundantly and widely applied in the manufacturing of polycarbonate plastics, epoxy resins, flame retardants, food containers and utensils, dental sealants, and protective coatings in food and beverage metal cans, baby bottles, and water supply pipes^6, 7^. Some studies reported that BPA can leach from these polymers and eventually enter the human body through dermal exposure and dietary intake; moreover, this substance can be detected in the environment and was identified as an environmental pollutant^8, 9^. Its extensive application induces a high risk of human exposure.

Alternative bisphenols (BPs) share similar structures and biological activities with BPA^6, 10^. These compounds, which consist of two phenolic rings joined together by a bridging carbon or other chemical structures, are also called BPA-related compounds and are used in some fields to replace BPA. Bisphenol B (BPB, 2,2-bis(4-hydroxyphenyl)butane)) and bisphenol E (BPE, 4,4′-ethylidenebisphenol) are bisphenol-type compounds, which are derivatives of BPA. These compounds are a low-cost BPA substitute that are commonly used in the polymer industry to manufacture phenolic resins and thus are commonly found in canned tomatoes^7^, soft drinks, and beers^11^. According to the current BP standard evaluation procedures, these bisphenol-type compounds have a moderate to slight toxicity and can modify natural endocrine functions by binding to the estrogen receptor, which consequently cause adverse effects, such as breast cancer, endometriosis, and infertility, on human health and wildlife^6, 12^. Similar to BPA, BPB presents endocrine-disrupting activities, specifically high estrogenic and anti-androgenic activities^13^. Cobellis *et al*.^14^ revealed strong evidence of the relationship between endometriosis and BPB exposure. Toxicological experiments by Chen *et al*.^6^ revealed that BPB exhibits highly acute toxicity. Moreover, BPB degrades at a slower rate than other BPs in aquatic environments^4^. A previous research showed that BPE is a typical environment- and endocrine-disrupting compound with estrogen-like activity; BPE is used in the production of phenolic resins and many consumer products, such as food containers, paper products, water pipes, toys, and medical equipment^6^. Thus, BPB and BPE are ubiquitous in the environment, exposing humans to BPB and BPE, which leach from into the food and/or saliva^14^. BPB and BPE have high lipophilicity and can thus accumulate in adipose tissues, leading persistent physiology toxicity, especially after prolonged exposure^14^.

Although some of the risks mentioned above require evaluation from medical trials or pharmacological experiments on animals and humans, these risks nevertheless cause perturbation in the public. As derivatives of BPA, BPB and BPE in the human blood sera and urine have been systematically tested and reported in recent years through toxicological action and detection methods^1, 14^. However, less attention has been paid to the protein-binding characteristics of BPB and BPE. These characteristics might provide useful information on the metabolism and toxicity of BPs and mechanism underlying the binding of BPs to specific proteins. Human serum albumin (HSA) is a principal extracellular protein with a concentration of 40 mg/mL or 0.6 mM in blood plasma and contains 50–60% of total plasma protein in humans^15, 16^. When EDs enter the human body, they are transported by HSA through the circulatory system. Zhang *et al*.^17^ preliminarily investigated some aspects of the interaction between BPA and HSA. Their report showed that BPA can bind to HSA molecules and alter the protein structure and influence the normal function of HSA. Thus, studying the interaction HSA with BPB and BPE is important.

The present work is a comprehensive *in vitro* study on the interaction of BPs with HSA using ^1^H saturation transfer difference nuclear magnetic resonance (STD-NMR), isothermal titration calorimetry (ITC), Fourier transform infrared (FT-IR), circular dichroism (CD), synchronous and 3D fluorescence spectroscopy, and molecular docking simulation. These techniques are complementary, and their findings were consistent with one another. STD–NMR was performed to determine whether BPs bind to HSA and the mechanism involved in the binding process. ITC was performed to obtain a comprehensive thermodynamic characterization of BPB–HSA and BPE–HSA systems. Furthermore, fluorescence competitive experiments combined with molecular docking simulation were conducted to locate the binding sites and types of binding forces involved in the binding process. The effects of BPs on local HSA conformation and the microenvironment of tryptophan (Trp) residue were also examined through FT-IR, CD, synchronous and 3D fluorescence spectroscopy.

## Results and Discussion

### Interactions between BPs and HSA

**Figure 1:**
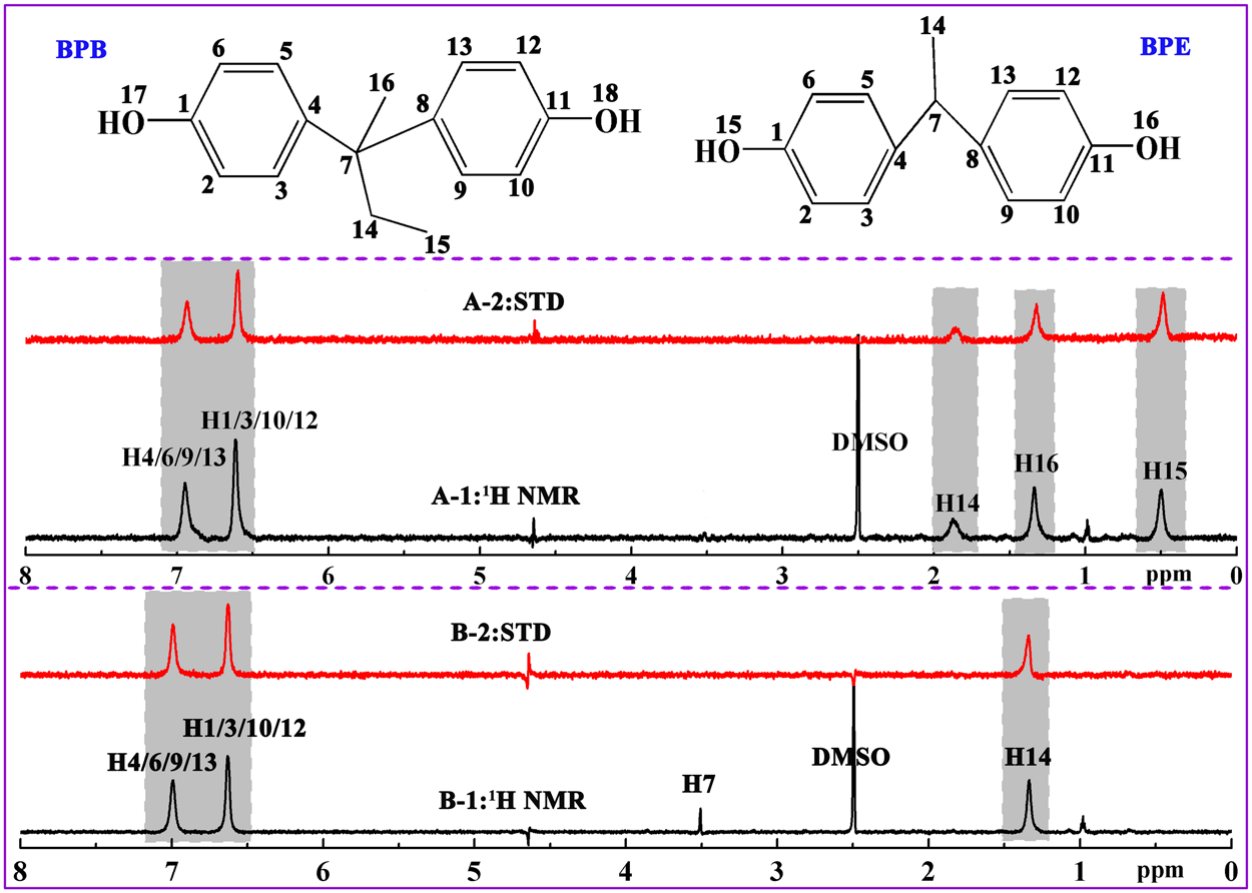
^1^H NMR spectrum **(A-1)** and STD-NMR spectrum **(A-2)** of BPB in the presence of HSA; ^1^H NMR spectrum **(B-1)** and STD-NMR spectrum **(B-2)** of BPE in the presence of HSA. Protein signals (1 ppm) corresponding to BPB and BPE are identified in the ^1^H NMR spectra. The top represents the BPB and BPE structures. [BPB/BPE] = 400 μM, [HSA] = 10 μM, pH = 7.4, T = 298 K.

STD-NMR is a ^1^H NMR technology that is based on nuclear Overhauser effect transference from the receptor to the ligand and is appropriate for observing biological binding events at the molecular level^18^. An STD spectrum involves subtracting on-resonance spectrum from the off-resonance spectrum. On-resonance spectrum is obtained by irradiating at a region of the spectrum that contains only the resonances of the receptor, whereas off-resonance spectrum is recorded without protein saturation^19^. Only the signals of ligands that received saturation transfer from the protein and whose intensities are affected by protein saturation are retained in the difference spectrum^19^. In the first experiment, we conducted ^1^H NMR and STD-NMR experiments, in which we used two BPs and an HSA to analyze their interaction. The ^1^H NMR and STD spectra of BP–HSA systems with a molar ratio of BPB/BPE to HSA of 40:1 are displayed in Fig. 1. All protons are relative to the corresponding chemical shift values, and some protons have the same chemical shift values because of the symmetry of their chemical environment, as shown in Fig. 1(A-1) and (B-1). The signal at δ = 1 ppm is not assigned in Fig. 1(A-1) and (B-1) because of the effect of protein signal. Clear STD signals are observed in all the BPB and BPE protons, indicating that protein saturation distributed onto the BPB and BPE, which bind to HSA under our experimental conditions.

The ligand hydrogen closest to the protein receives the most intense magnetization transfer and displays the largest relative intensity^18–20^. The binding epitopes were identified by calculating the relative STD (%) effect (*R*_STD_), which reflects the relative amount of saturation received by the protein via cross-relaxation and the relative proximity of proton to the protein-binding site. For the calculation of *R*_STD_, the STD signal with the largest integral value was set to 100%, and all other STD signals were calculated accordingly^20, 21^. The calculated *R*_STD_ values for the hydrogen protons of BPB and BPE clearly showed that the BPs have different affinities towards HSA, as summarized in supplementary Table S1. BPB and BPE showed a nearly identical *R*_STD_ values, that is, H1/3/10/12 protons exhibited 100% *R*_STD_, followed by H4/6/9/13 protons. Thus, the BPs bind to HSA in a similar pattern, in which the hydrogen atoms on the phenolic rings of BPB and BPE are in a close contact with HSA interacting sites. For BPB, the hydrogen atoms in methylene (H14) provide the lowest *R*_STD_ value (nearly 25%), suggesting that methylene is oriented away from the interacting site or in a position in which it cannot receive abundant magnetization transfer from HSA because of space steric hindrance.

### Thermodynamic parameters and binding modes

**Figure 2:**
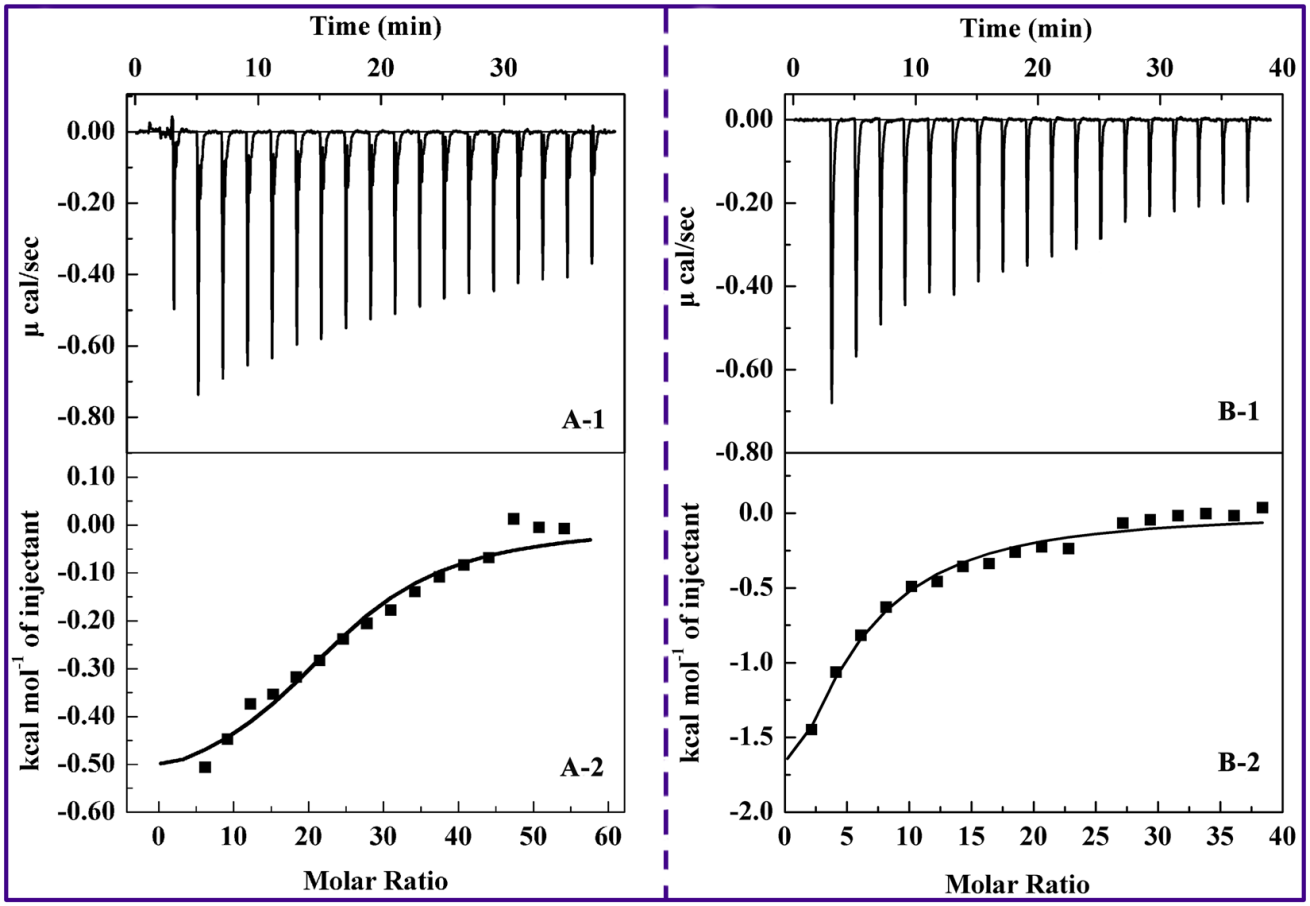
**(A-1)** Raw data for the titration of 3 mM BPB with 10 μM HSA at pH 7.4, showing that the calorimetric response as successive injections of BPB is added to the sample cell. **(A-2)** Integrated heat profile of the calorimetric titration shown in panel A-1. **(B-1)** Raw data for the titration of 3 mM BPE with 20 μM HSA at pH 7.4, showing that the calorimetric response as successive injections of BPE is added to the sample cell. **(B-2)** Integrated heat profile of the calorimetric titration shown in panel B-1.

In contrast to conventional methods, such as fluorescence, UV-visible absorption, and electrochemical method, ITC can directly measure evolving heat during a reaction without requiring chemical modification or immobilization of reactants^22, 23^. The thermodynamics of protein–ligand interaction measured by ITC is a global development. Thus, the binding affinity and associated thermodynamics of BPs with HSA were investigated through ITC. Representative calorimetric titration profiles of the interaction between BPB/BPE and HSA at 298 K are shown in Fig. 2. The single injection of BPs into an HSA solution can be reflected by each peak in the binding isotherm, as displayed in panels A-1 and B-1. The integrated plot of the amount of heat liberated per injection as a function of the molar ratio of BPB/BPE to HSA is shown in panels A-2 and B-2. In Fig. 2A and B, BPB/BPE-HSA curves show a regular shape with only one evident interaction jump. The curves are typical of thermodynamics controlled ligand–protein interactions, with relatively decreasing exothermic peaks upon BPB and BPE addition. The association constant (*K*), enthalpy change (Δ*H*), and entropy change (Δ*S*) were directly obtained from the fitted results. The free energy change (Δ*G*) can be calculated from the values of Δ*H* and Δ*S* according to the following equation:

1 $${\rm{\Delta }}G={\rm{\Delta }}H-T{\rm{\Delta }}S$$

The results in Table 1 suggested that the two BPs interact with HSA through the same binding mode. Moreover, the *K* values suggested that HSA has moderate affinity to BPB and BPE, because the documented *K* value^4^ of noncovalent association of HSA with compounds is mostly in the range of 10^4^–10^6^ M^−1^ ^24^. The *K* values also indicated that the binding of BPE to HSA is relatively stronger than that of BPB. This result may be attributed to the ethyl group substitute for benzyl hydrogen proton. The steric hindrance of BPB results in the decrease in the number of molecules inserted into the binding pocket of HSA. The results are consistent with those of STD-NMR. Several types of noncovalent interactions, such as hydrogen bonds, van der Waals forces, and hydrophobic and electrostatic interactions, are involved protein–ligand interactions^25^. The type of binding force can be determined by ∆*H* and ∆*S* according to the theory of Ross and Subramanian^26^. In particular, Δ*H < *0 or Δ*H* ≈ 0 along with Δ*S* > 0 correspond to electrostatic force and hydrogen bonds. Furthermore, Δ*H* < 0 and Δ*S < *0 refer to Van der Waals interactions and hydrophobic interactions, and Δ*H* > 0 and Δ*S* > 0 indicate hydrophobic force. Thus, the negative Δ*H* and positive Δ*S* values of BPB-HSA and BPE-HSA systems confirmed that electrostatic interactions and hydrogen bonds play a major role in binding reactions; however, other forces, such as hydrophobic interactions, cannot be excluded^27–29^. The reactions are consistent with the “enthalpy–entropy compensation effect”, in which the enthalpy drop due to the deformation of hydrogen bonds is counter-balanced by the entropic penalty due to the burial of involved groups in the interaction^30^. This effect is frequently found in other ligand–protein interactions^31, 32^. The free energy (Δ*G*) was calculated from Eq. (1), and the results showed that the binding of BPB or BPE to HSA is spontaneous.

**Table 1:** Thermodynamic parameters for interaction of BPB and BPE with HSA obtained from ITC at pH 7.4.

BPs	*K* (×10^4^ M^−1^)	Δ*H* (kcal mol^−1^)	Δ*G* (kcal mol^−1^)	Δ*S* (cal mol^−1^ K^−1^)
BPB	2.80 ± 0.92	−0.57 ± 0.05	−6.06	18.4
BPE	7.79 ± 2.46	−4.79 ± 2.72	−5.65	2.88

### Site-selective binding of BPs on HSA

Human serum albumin is a single-chain and nonglycosylated globular protein consisting of 585 amino acids and stabilized by 17 disulfide bridges^33^. Endogenous and exogenous compounds in the blood stream have two primary ligand binding sites for HSA, which are called site I and site II and located in the hydrophobic subdomains IIA and IIIA, respectively^34, 35^. Fluorescence competitive studies with two effective binding competitors, DNSA and DNSS, were conducted to evaluate whether the binding sites of BPB and BPE are similar (or overlap) to those already confirmed for other ligands. Given that DNSA and DNSS can bind at sites I and II, respectively, they were used as fluorescence probes by Sudlow *et al*.^35^ to identify the binding site of multiple small molecules, such as warfarin, ibuprofen, naproxen, and phenprocoumon. Sudlow *et al*.^35^ proposed a rapid and simple method for the determination of the ligand binding region based on the competition for protein binding sites. According to Sudlow *et al*.^35^, the fluorescence of DNSA/DNSS-HSA systems can be measured in the absence and presence of different ligand concentrations, and the percentage (*I*) of probe displacement can be calculated as follows:

2 $$I=\frac{{F}_{1}}{{F}_{2}}\times 100 \% $$

where *F*_1_ and *F*_2_ represent the fluorescence intensity of probe–HSA in the presence and absence of the ligand, respectively.

**Figure 3:**
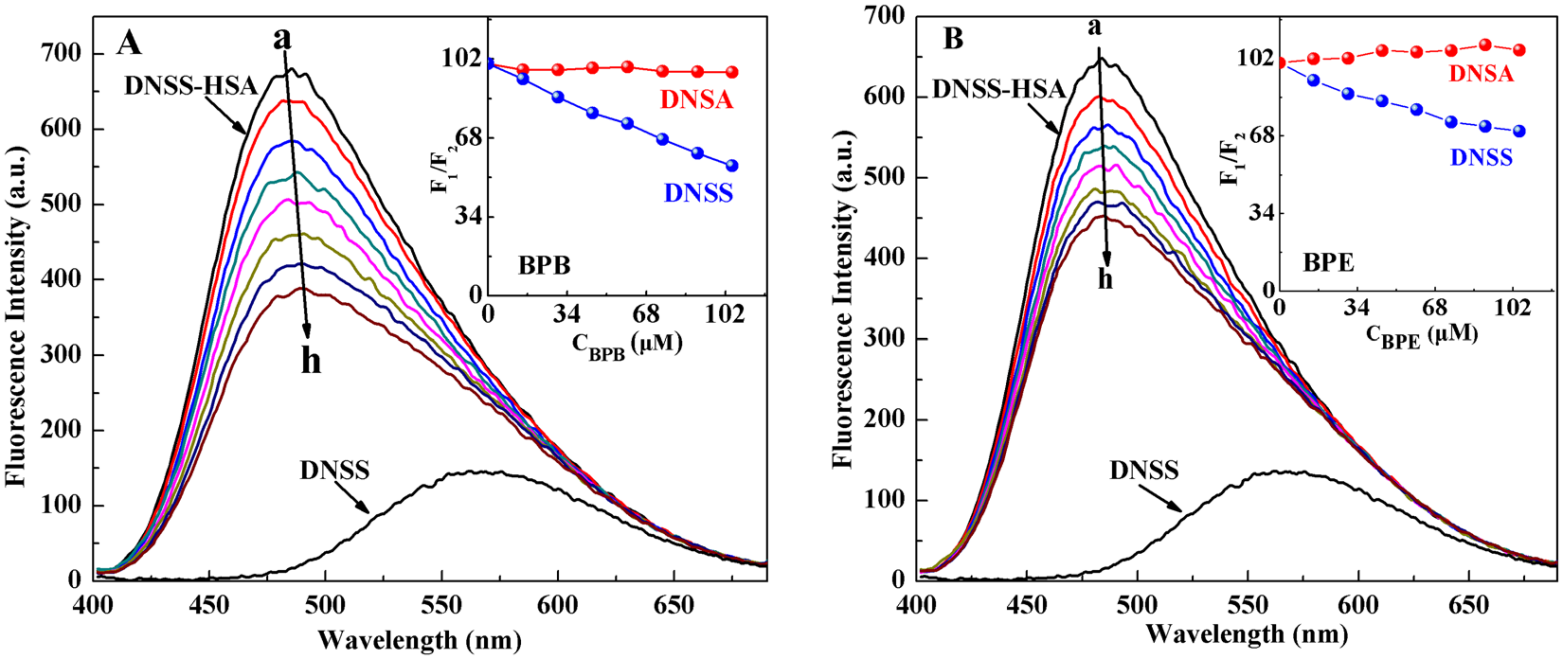
Fluorescence spectra of DNSS-HSA in the absence and presence of BPB (**A**) and BPE (**B**). The HSA concentration was 2 μM. The concentration of DNSA/DNSS was 20 μM. The BPB/BPE concentrations were 0 (a), 15 (b), 30 (c), 45 (d), 60 (e), 75 (f), 90 (g), and 105 (h) μM. Insert portion shows the effect of BPB/BPE on the fluorescence of HSA-DNSA/DNSS systems.

A decrease in the fluorescence intensity of the probe–HSA complex can be interpreted as a displacement of the probe from its binding site as induced by the added ligand^36^. With the addition of HSA into DNSS, the maximum emission wavelength of DNSS has an obvious blue shift, and the fluorescence intensity is significantly higher than that of without HSA (Fig. 3). BPB/BPE was added subsequently to the solution containing DNSS-HSA and the fluorescence was recorded. As shown in Fig. 3, BPB and BPE reduced the fluorescence intensity of the DNSS–HSA complex in a concentration-dependent manner. In the presence of 105 μM BPB/BPE, the fluorescence intensities of DNSS were reduced to approximately 55% and 69% of the initial intensity, respectively. This suggested that both DNSS and BPB/BPE compete for site II in HSA and further, DNSS was displaced by BPB and BPE from HSA. As the free DNSA increased in solution, leading a little red shift in the emission maximum. However, the fluorescence intensity of the DNSA–HSA complex was barely influenced by BPB/BPE even when large concentration of BPB/BPE was added, indicating that either BPB/BPE does not bind to site I or BPB/BPE has weaker binding capacity to site I than DNSA. Further information about binding sites was obtained from the molecular docking simulations.

### Analysis of Molecular Docking Results

**Figure 4:**
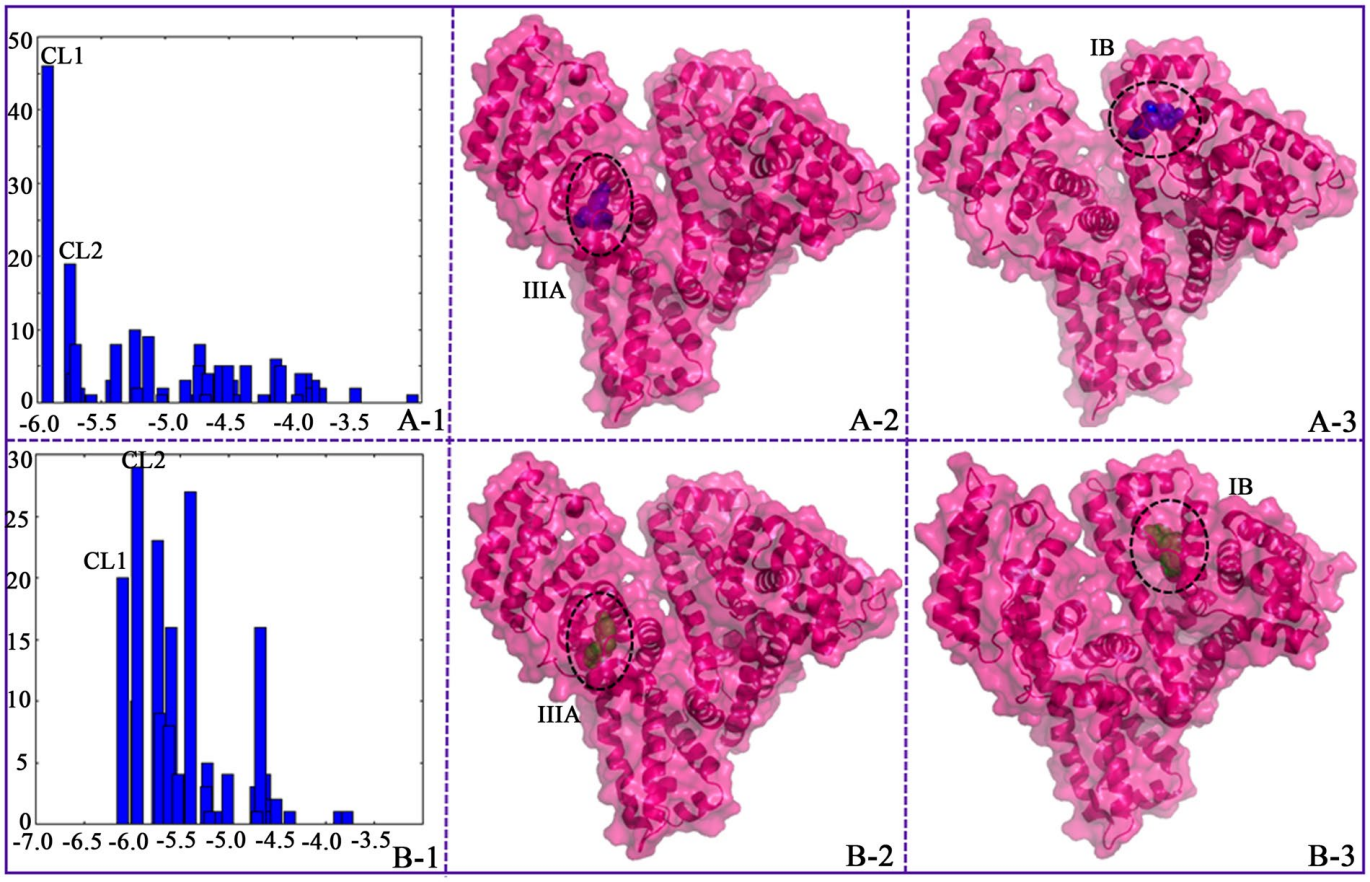
Cluster analyses of the AutoDock docking runs of BPB–HSA system **(A-1)** and BPE–HSA system **(B-1)**. Docked configuration of BPB–HSA and BPE–HSA systems with the lowest binding energy in CL 1 (**A-1** and **B-1**), and CL 2 (**A-2** and **B-2**), respectively.

The potential binding locations of BPs for proteins were further investigated through molecular docking simulation. A systematic search by AutoDock strategy can examine the entire surface of protein for the binding sites of BPs and corroborate the experimental results. Cluster analysis was performed using a root mean square deviation tolerance of 2.0 Å, as shown in Fig. 4(A-1) and (B-1). Consequently, four different binding locations were obtained from 200 docking runs for BPB, whereas five different binding locations were obtained for BPE. For the BPB-HSA system, the first cluster (CL1, 46 out of 200 conformations) for the hydrophobic subdomain IIIA of HSA had the lowest energy. From the docking simulation, the observed energy change of binding for the BPB-HSA complex was calculated to be −5.92 kcal mol^−1^, which is in good agreement with the determined value from ITC (−6.06 kcal mol^−1^). In Fig. 4(A-3), the analysis of the docking disposition of the conformers belonging to CL2 supported the possible interaction of BPB with the subdomain IB of HSA and revealed an estimated docking energy of approximately −5.75 kcal mol^−1^. Although CL2 was found to have 19 distinct conformers, this cluster is not the highest populated and most energetically favorable. Therefore, the hydrophobic subdomain IIIA of the protein is the favorable binding region for BPB. The result indicated that the model docking poses and scores support the experimental findings from the fluorescence competitive studies. For the BPE–HSA system, CL1 is the most energetically favorable conformational cluster with an estimated docking energy of approximately −6.10 kcal mol^−1^. The global views of the favorable docked configuration are displayed in Fig. 4(B-2), and BPE is possibly located in the hydrophobic cavities in the subdomain IIIA of site II. A total of 29 distinct conformational models were observed for the most populated cluster (CL2). The result revealed that the BPE might bind to the subdomain IB of HSA with a docking energy of −5.95 kcal mol^−1^. However, CL2 is not the most energetically favorable for the subdomain IB. Based on the findings from the fluorescence competitive experiments, BPE binds preferentially to ligand binding site II (subdomain IIIA) of HSA.

**Figure 5:**
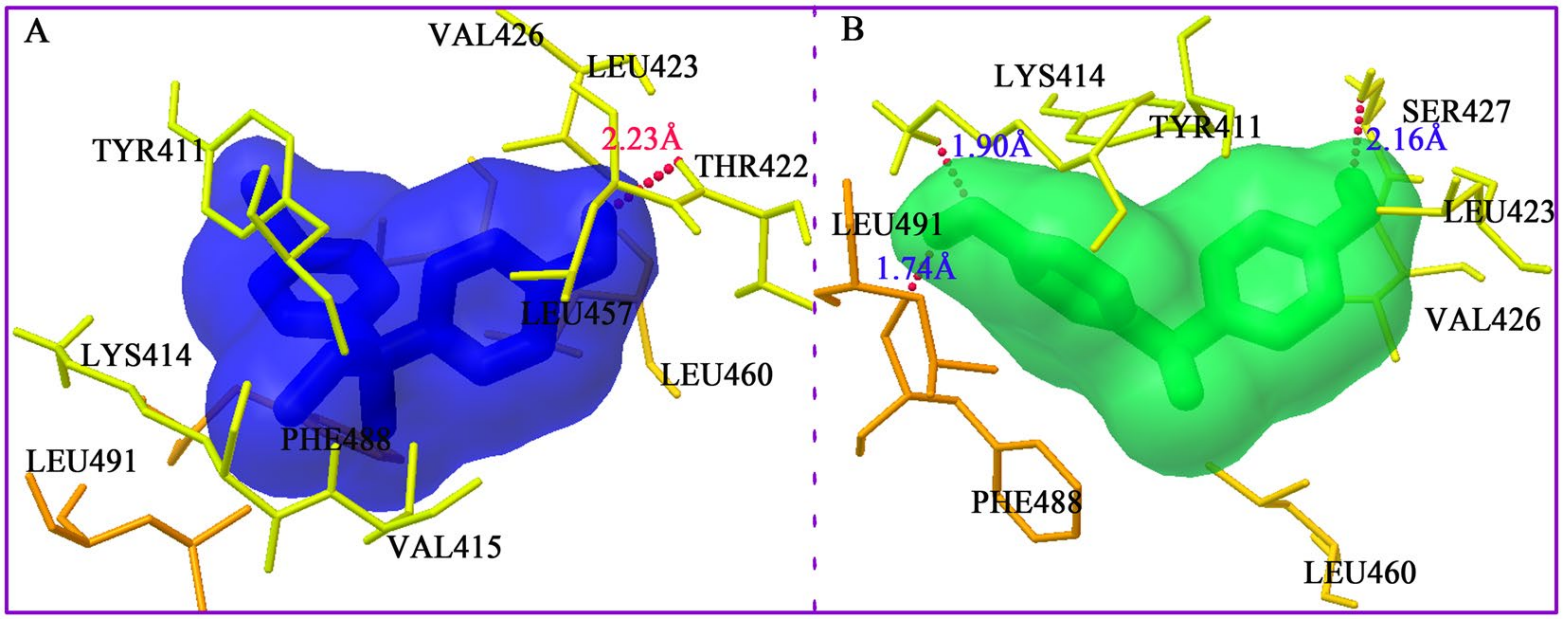
Best docked results of the HSA–BPB (**A**) and HSA–BPE systems (**B**). Ligands (BPB and BPE) are shown in stick representation and colored as follows: BPB, blue; BPE, green.

The most energetically favored cluster for the subdomain IIIA in binding site II of HSA was used for further binding-orientation analysis (Fig. 5). Figure 5A and B show that BPB and BPE interact with the TYR411, LYS414, VAL (415, 426), LEU (423, 460, 491), and PHE488 residues of subdomain IIIA through hydrophobic interactions. Furthermore, a number of specific hydrogen bonds can be observed, because several correlative residues near the ligands play an important role in the binding of BPB and BPE molecules via hydrogen bonds. Hydrogen bonding interactions were observed between the H atom on hydroxyl group of BPB and THR422 of HSA with a hydrogen bond distance of 2.23 Å. These hydrogen bonds include the bond between SER427 and H atom on the hydroxyl group of BPE and those between LYS414 and PHE488, as well as other hydroxyl groups of BPE, with distances of 2.16, 1.90 and 1.74 Å, respectively. The hydroxyl group on the phenyl moieties of BPB and BPE interact with HSA through hydrogen bonding, and this finding agrees with the STD-NMR data, which indicated that phenyl moieties receive more saturation from the HSA.

### Investigations of HSA conformational changes

#### Fourier transform infrared spectroscopy

**Figure 6:**
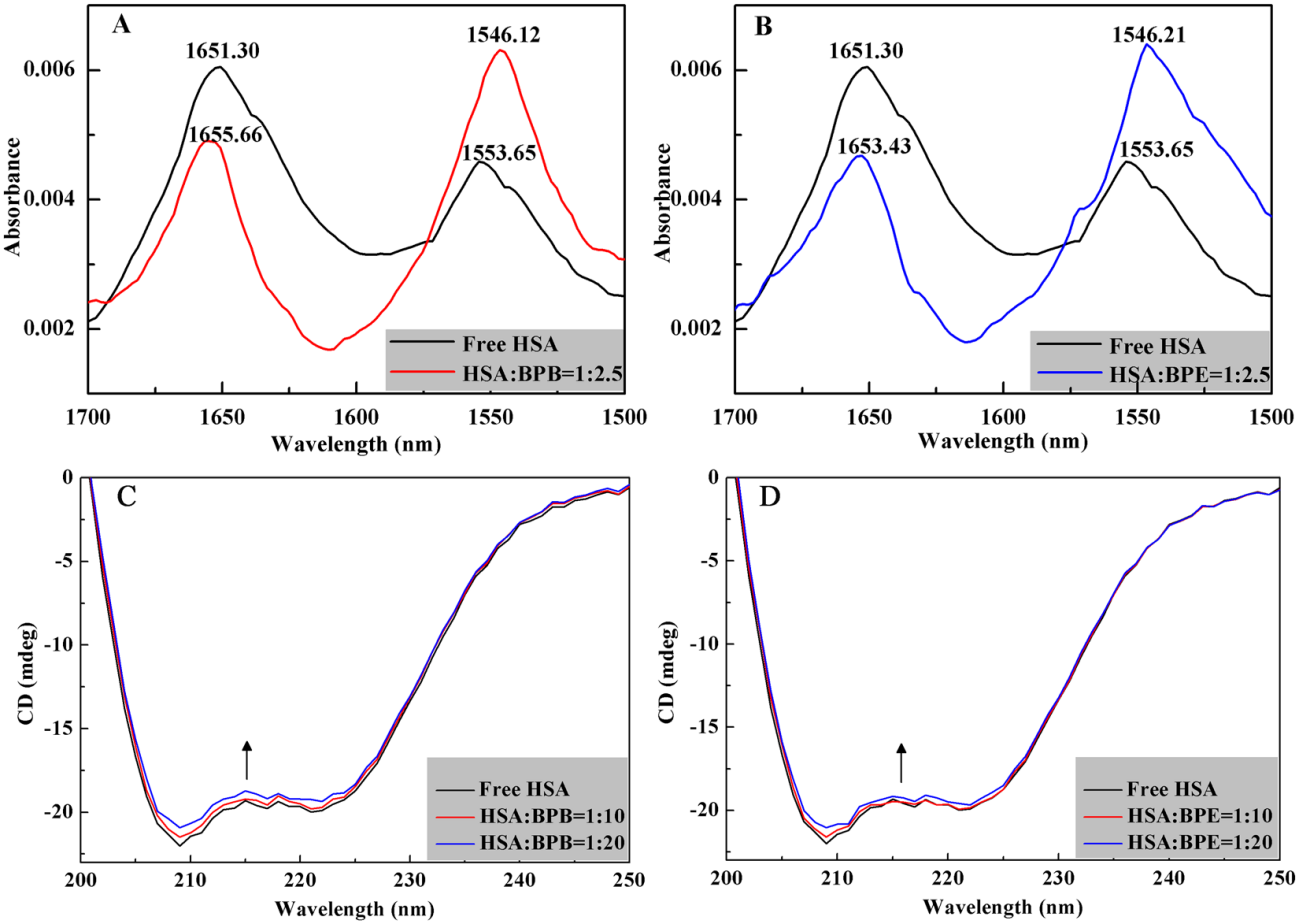
FT-IR spectra of HSA-BPB **(A)** and HSA-BPE **(B)** systems. The concentrations of HSA and BPs were 0.19 mM and 0.47 mM, respectively. CD spectra of free HSA (2 μM) and its complexes with BPB **(C)** and BPE **(D)**. The molar ratios for [HSA]/[BPs] were 1:0, 1:10, 1:20.

Firstly, the conformation of HSA affected by BPs was investigated through FT-IR spectroscopy. The IR spectrum of protein exhibited a number of amide bands representing the different vibrations of peptide moiety. It could be seen from Fig. 6A and B that HSA exhibited two main amide bands (amides I and II bands) around 1651.30 cm^−1^ (mainly C = O stretching) and 1553.65 cm^−1^ (C-N stretching coupled with N-H bending) related with the secondary structure of protein^37^. After adding BPB and BPE, the peak positions of the amides I and II bands in the two difference spectra were observed to shift with a simultaneous change in the relative intensity. In comparing with the spectrum of HSA, the spectra of HSA-BPs systems exhibited a slight wavelength red shift (from 1651.30 cm^−1^ to 1655.66 and 1653.43 cm^−1^ for BPB and BPE, respectively) and an obvious wavelength blue shift (from 1553.65 cm^−1^ to 1546.12 and 1546.21 cm^−1^ for BPB and BPE, respectively) in the amides I and II bands. These results indicated that BPB and BPE interacted with both the C = O, C-N, and N-H groups in the polypeptides of HSA that results in conformational changes in the secondary structure of HSA^37, 38^.

#### Circular dichroism spectroscopy

Circular dichroism (CD) spectroscopy is another effective method for the characterization of protein structures and evaluation of conformational changes in proteins interacting with ligands^37^. The far-UV CD spectrum of HSA exhibited a typical shape, which corresponded to an α-helix-rich secondary structure and revealed two negative bands at 208 (π−π*) and 220 nm (n−π*)^37, 39, 40^. The CD spectra of free HSA and its complexes were measured to confirm the influence of BPB/BPE binding on secondary HSA structure (Fig. 6C and D). The position and shape of peak in the CD spectra are similar before and after adding BPB/BPE, suggested that the HSA structure is predominantly α-helix. However, the CD intensity slightly decreased after adding BPB/BPE, indicating the reduced α-helicity of native HSA. The % α-helicity of HSA was estimated from the *MRE* values at 208 nm through the following equation^40^:

3 $${\rm{\alpha }}-{\rm{helix}}\,( \% )=\frac{-{MR}{{E}}_{208}-4000}{33000-4000}\times 100$$

The α-helical content of free HSA (51.06%) is in good agreement with those mentioned in previous reports^40, 41^. This percentage decreases to 47.87% and 49.49% upon the addition of BPB and BPE with a molar concentration ratio of 1:20. These data indicated that BPB has greater effect on the secondary structure of HSA than BPE. The different effects of the two compounds on the α-helical content of HSA might be associated with the bound conformations of these interacting BPs. The decreasing α-helix content indicated that the binding of BPB/BPE with HSA induces a slight unfolding of the constituent polypeptides, changes in secondary HSA structure. Thus, the results showed that the interaction between BPB/BPE and HSA causes the conformation changes of HSA.

#### Synchronous fluorescence spectroscopy

Synchronous fluorescence is a simple and sensitive method for the characterization of the interaction between a fluorescence quencher and protein. This technique can provide characteristic information on the polarity change around the chromophore micro-environment^15, 16^. The advantages of this method are spectral simplification, reduction of the spectral bandwidth, and prevention of different perturbing effects. According to the theory of Miller^42^, synchronous fluorescence spectra are achieved by simultaneously scanning the excitation and emission monochromators and only showing the Tyr and Trp residues of HSA when the wavelength interval (Δλ) is 15 nm and 60 nm, respectively.

The synchronous fluorescence spectra of HSA with various amounts of BPB and BPE recorded at Δλ = 60 nm are shown in supplementary Fig. S1. The Tyr emission spectra were not presented, because the maximum emission wavelength of HSA and that of BPB/BPE were close to each other when Δλ was set at 15 nm. The overlapping peaks increased the erroneous interpretation of the results. Therefore, following quenching of the fluorescence intensities of Trp for HSA on addition of the BPs (0–120 μM) can conclude whether the BPB/BPE intercalate to the protein at the site close to Trp, and the changes of HSA conformation would be estimated. Compared with ITC, no saturation phenomenon was observed in the spectroscopy experiments even the molar ratio of BPs/HSA up to 60 times. This phenomenon is common in ligand-protein interaction^22, 32, 43^. Ishtikhar *et al*.^32^ have explained that the differences of binding affinity obtained by ITC and fluorescence spectroscopy may be due to the location of ligands and fluorophore in the later. Fluorescence competitive experiments coupled with docking studies revealed that BPs preferentially bound to site II in the hydrophobic subdomains IIIA of HSA and large numbers of BPs were far from the single Trp214 in HSA. Thus, in order to observe the change in the microenvironment of Trp residue in HSA, a large ratio of moles in BPs and protein were selected. As seen from the supplementary Fig. S1A, the position of the maximum emission wavelength showed significant blue shifts (4 nm) at increasing BPB concentrations, suggesting that the conformation of HSA was changed by BPB, hydrophobicity around Trp residues increased, and polarity decreased^40^. No obvious shift in the emission wavelength of Trp was observed in the BPE–HSA system (see the supplementary Fig. S1B), indicating that BPE has minimal effect on the micro-environment of the Trp residues in HSA. These findings are consistent with the above CD results.

#### Three-dimensional fluorescence spectroscopy

**Figure 7:**
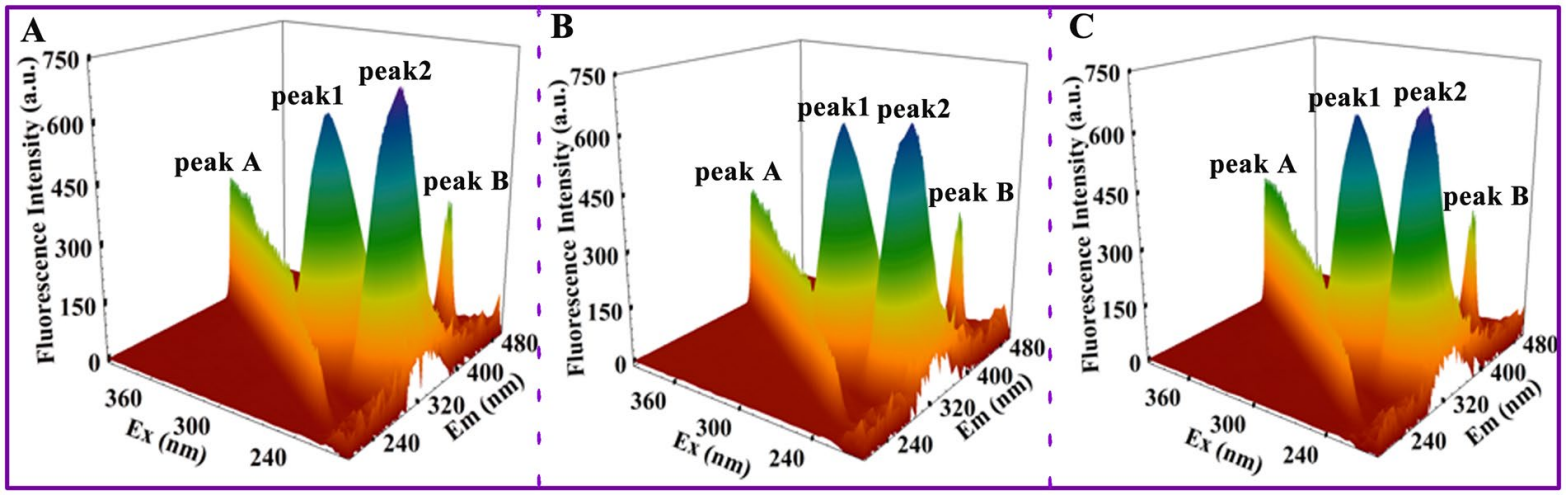
3D Fluorescence spectra of (**A**) HSA, (**B**) BPB–HSA system, and (**C**) BPE–HSA. [HSA] = 2 μM, [BPB/BPE] = 10 μM, pH = 7.4, T = 298 K; λ_ex_ = 200–400 nm, λ_em_ = 200–500 nm.

Three-dimensional fluorescence spectroscopy can be used to investigate the variation in HSA conformation. As presented in Fig. 7 and supplementary Table S2, the fluorescence peaks A and B represent the first-order Rayleigh scattering peak (λ_ex_ = λ_em_) and second-order scattering peak (2λ_ex_ = λ_em_), respectively^37, 41^. Peak 1 (λ_ex/em_ = 280/337 nm/nm) mainly reveals the spectral behavior of Trp or Tyr residue of HSA, whereas fluorescence peak 2 (λ_ex/em_ = 225/333 nm/nm) exhibits the spectral behavior of polypeptide backbone structures^37^. The fluorescence intensities of these peaks are correlated to the polarity of the microenvironment and the secondary structure of the protein. The band intensity of peak 2 decreased after the titration of BPB/BPE, and the magnitude of Stokes shift (2 nm) decreased (supplementary Table S2). This findings indicated the altered polypeptide structures of HSA upon the binding of BPs. Moreover, the intensity of fluorescence peak 2 in the BPB–HSA system was lower than that in the BPE–HSA system, indicating that more polypeptide structures are changed after the addition of BPB. For peak 1, fluorescence intensity increased after BPs concentration were increased. The possible reasons for this behavior are provided in the foregoing synchronous fluorescence spectroscopy. The fluorescence intensity of peak A increased after BPE addition, possibly because of the increase in the diameter of HSA after binding with BPE. Increasing the diameter of HSA can enhance the Rayleigh scattering peak^41, 44^. Changes in 3D fluorescence spectra characteristics are similar to the synchronous fluorescence results, which revealed that some micro-environmental and conformational changes in HSA are slightly altered in the presence of BPs.

## Conclusions

This paper presents the first detailed investigation on the interaction between BPs and HSA using experimental and computational approaches. The results confirm that although both BPs bind preferentially to the subdomain IIIA in the binding domain of the protein, the magnitude of interaction differs significantly. The STD-NMR results, assisted by docking calculations, reveal that the compounds BPB and BPE bind to HSA in a similar pattern, in which the phenolic rings are in a close contact with HSA binding site. The association constant and thermodynamic parameters were confirmed by ITC. The negative values of Δ*G* were associated with spontaneity of interaction. The negative Δ*H* and positive Δ*S* revealed that the two interactions were mainly driven by hydrogen binding and electrostatic forces. The binding affinity of BPB to HSA was weaker than that of BPE because of the steric hindrance of the ethyl group on the BPB. Displacement of DNSS competitively by BPB/BPE revealed that BPB and BPE can bind at site II of HSA. This result can be corroborated further by molecular docking studies. FT-IR, CD, synchronous and 3D fluorescence spectra of HSA in the absence and presence of BPs were measured to investigate the conformational change of protein, which indicated that the protein secondary structure and microenvironment were slightly affected by BPB and BPE. In conclusion, this study presents fundamental but valuable information on the interaction between BPs and HSA. Our findings can provide important insights into the binding mechanisms between EDs and proteins.

## Materials and Methods

### Materials and chemicals

Fatty acid-free HSA was purchased from Sigma Aldrich (Milwaukee, USA) and used without further purification. BPB (purity > 99%), BPE (purity > 99%), deuterium oxide (D_2_O, 99.9% purity), dimethyl sulfoxide-d6 (DMSO-d6), and 5-dimethylaminonaphthalene-1-sulfonamide (DNSA) were purchased from J&K Scientific, Ltd. (Beijing, China). Dansylsarcosine (DNSS) was obtained from Heowns Biochemical Technology Co., Ltd. (Tianjin, China). All other reagents were of analytical grade and used without further purification.

### STD NMR measurements

STD NMR experiments were conducted on a Varian 700 MHz Inovas pectrometer operating at 298 K with VNMRJ software (version 2.1B). The stock solutions of BPB/BPE and HSA were prepared in DMSO-d6 and PBS (0.01 M in D_2_O, pH = 7.4), respectively. Different amounts of BPB/BPE and HSA were mixed and diluted with PBS to obtain a final solution containing 10 μM HSA and 400 μM BPB/BPE. A train of Gaussian-shaped pulses of 50 ms and with central frequency between on-resonance saturation of −0.5 ppm and off-resonance saturation of 34 ppm were applied for the STD measurements. The total number of scans was 1024, and 16 ppm spectral widths were typically used for the ^1^H STD spectra. A sweep width of 8389.26 Hz, number of transients of 256, acquisition time of 1 s, radiation power of 8 dB, irradiation time of 2 s, and around B1 field strength of 100 Hz were set to obtain all spectra, which were further processed and analyzed using ACD/CNMR software (Advanced Chemistry Development, Inc., version 11.0). The binding epitope of BPB and BPE calculations were then made by comparing the individual peak integrals, and normalizing others with respect to this, which was earmarked for 100%.

### ITC analysis

An isothermal titration calorimeter (CN-ITC200; MicroCal, USA) was used to titrate HSA with BPB/BPE to obtain thermodynamic information at 298 K. To prepare HSA solution, the dialysis tubing was filled with HSA at a proper concentration in PBS buffer-containing DMSO and was incubated at 277 K for 24 h. The final DMSO concentration did not exceed 5% by volume. The concentration of the HSA was determined on a UV-1800 Shimadzu ultraviolet (UV)-spectrophotometer (Japan) using the extinction coefficient ε_280_ = 35700 mol^−1^ L cm^−1^ ^25^. In order to avoid interference of DMSO, BPB and BPE were directly dissolved in dialysate. All solutions were properly degassed before the ITC sample chamber was loaded to prevent bubble formation. The concentrations of BPB and BPE solutions were both 3 mM, but those of HSA solutions were 10 and 20 μM. After being subjected to temperature equilibration, the solutions were successively injected into the reaction cell in 2 μL increments at 120 s intervals and with stirring at 750 rpm to ensure thorough mixing. Raw data were obtained as a plot of heat flow (μcal) against time (minutes) are then integrated peak-by-peak and normalized to obtain a plot of observed enthalpy change per mole of injection against the molar ratio (BPs/HSA). The raw data peaks were transformed using the instrument’s software, and related parameters were calculated using MicroCal Origin software.

### Fluorescence spectra measurements

A Cary Eclipse fluorescence spectrophotometer (Varian, USA) equipped with a 1-cm quartz cell was used to identify the main binding sites of BPB/BPE for HSA. Then, DNSA and DNSS, two known binders of site I and site II of HSA, were used as the fluorescence probes. The mixed solution contained HSA (2 μM) and DNSA/DNSS (20 μM) and was subsequently added with different concentrations of BPB/BPE. The excitation wavelengths for DNSA and DNSS were 326 and 350 nm, respectively. Synchronous fluorescence spectra were recorded at Δλ = 15 and 60 nm. The HSA concentration was fixed at 2 μM in a quartz cell and the concentrations of BPB and BPE were varied from 0 to 120 μM by successive additions. The spectra were recorded in the wavelength range of 200–350 nm and 220–380 nm, for Δλ = 15 and 60 nm, respectively. The 3D fluorescence spectra of HSA (2 μM) and HSA–BPB/BPE solutions (molar ratio, 1:5) were scanned using an excitation wavelength ranging from 200 nm to 400 nm with 5 nm increments. The emission spectra were monitored between 200 and 500 nm.

### Molecular docking studies

All the docking simulations were performed using AutoDock Version 4.2.5.1 program package and AutoDock Tools (ADT) Version^29, 41^ to identify potential ligand binding locations. The crystal structure of HSA was obtained from the Protein Data Bank (PDB ID: 1H9Z). The structure was optimized by removing water, adding and assigning Kollman united-atom partial charges, and adding polar hydrogen atoms. The 3D ligand structures of BPB and BPE were obtained from PubChem (PubChem CID: 66166 and 608116). Blind docking with grid sizes of 126, 92, and 116 along the X-, Y-, and Z-axes, respectively, and grid spacing of 0.615 Å was conducted to determine all the binding sites in HSA. The center of the grid was set to 25.033, 9.563, and 18.684 Å. AutoGrid was used to generate the grid map for various atoms of the ligand and receptor. Lamarckian genetic algorithms were used to simulate the interaction between BPB/BPE and HSA and to describe their relationship through translation, orientation, and conformation of the ligands in AutoDock. Global optimization started with 200 runs in 250 000 energy evaluations and a maximum of 27 000 generations. Other parameters were set to default protocol settings. The docking model with the lowest binding free energy was selected to analyze the final conformation.

### FT-IR spectroscopy

The FT-IR spectra of HSA in the presence and absence of BPB and BPE were recorded on a Nicolet-6700 FT-IR spectrometer (Thermo, USA) equipped with a smart OMNI-sampler accessory. All spectra were obtained at 2000–1000 cm^−1^ by averaging 128 scans with a resolution of 4 cm^−1^ at room temperature. HSA concentration was fixed at 0.19 mM, whereas that of SOF was 0.47 mM. For the FT-IR spectra of HSA (in free form), the absorbance of buffer solution was firstly measured, and then digitally subtracted from that of the protein solution to get the spectrum of the protein alone. For the HSA–BPs systems in buffer solution (in bound form), the free BPB and BPE solution were recorded and digitally subtracted to get the spectra of complexes.

### CD spectra analysis

The conformational effect of BPB/BPE on HSA was investigated using a Chirascan CD spectrometer (Applied Photophysics Ltd., Leatherhead, U.K.) equipped with Peltier temperature control unit. The measurements were carried out at 298 K using quartz cuvette with a path length of 0.1 cm. HSA was diluted in PBS and the molar ratio of HSA to BPB/BPE was maintained at 1:0, 1:10 and 1:20 in which the concentration of HSA was 2 μM. The spectra of HSA and its ligand complex were obtained at 260–195 nm wavelengths, each of which had a step size of 1 nm, band width of 1 nm, and response time of 0.5 s. Each spectrum was the average of three successive scans and was corrected by PBS. The results were expressed as mean residue ellipticity (*MRE*) in deg cm^2^ dmol^–1^. *MRE* was defined as follows^37, 45^:

4 $$MRE=\frac{Intensity\,of\,CD\,(m\,\deg )\,at\,208\,nm}{{C}_{p}nl\times 10}$$

where *C*_*p*_ is the molar concentration of the protein, *n* is the number of amino acid residues (585 for HSA^37, 45^), and *l* is the path length of the cuvette.

## Electronic supplementary material

supplementary Information
