# Supplementary material for: Binding modes of environmental endocrine disruptors to human serum albumin: insights from STD-NMR, ITC, spectroscopic and molecular docking studies
Source: Sci Rep. 2017 Sep 11;7:11126. doi: 10.1038/s41598-017-11604-3 (PMC5593971; doi:10.1038/s41598-017-11604-3)
Supplement: Supplementary file 1 — supplementary Information [file 41598_2017_11604_MOESM1_ESM.pdf]

Supplementary Information

Binding modes of environmental endocrine disruptors to human serum albumin: insights from STD-NMR, ITC, spectroscopic and molecular docking studies

Hongqin Yang[1], Yanmei Huang[1], Jiuyang Liu[2], Peixiao Tang[1*], Qiaomei Sun[1], Xinnuo Xiong[1], Bin Tang[1], Jiawei He[1], Hui Li[1*]

[1]College of Chemical Engineering, Sichuan University, Chengdu 610065, China.

[2]School of Life Sciences, University of Science and Technology of China, Hefei 230026, China.

[*]These authors contributed equally to this work.

Correspondence and requests for materials should be addressed to P.-X.T (email: tangpeixiao@126.com) or H.L. (email: lihuilab@sina.com)

Table S1. Relative degrees of saturation of protons in STD-NMR spectra for BPB and BPE. The proton of H1/3/10/12 with the strongest STD effect was assigned with a value of 100%.

Hydrogen	H1/3/10/12	H4/6/9/13	H14	H15	H16
BPB-R_{STD}	100	61	25	59	55
BPE-R_{STD}	100	73	54	-	-

Table S2 Three-dimensional fluorescence spectral parameters of HSA alone and in the presence of BPB/BPE.

System	Peak No.	Peak position [λ_{ex} / λ_{em} (nm/nm)]	Δλ (nm)	Intensity
HSA	A	280/280→350/350	0	212.99→307.99
	1	280/338	58	621.95
	2	225/332	107	710.67
HSA–BPB	A	280/280→350/350	0	192.86→300.76
	1	280/337	57	633.01
	2	225/330	105	662.56
HSA–BPE	A	280/280→350/350	0	194.09→333.07
	1	280/333	53	647.29
	2	225/334	109	692.05

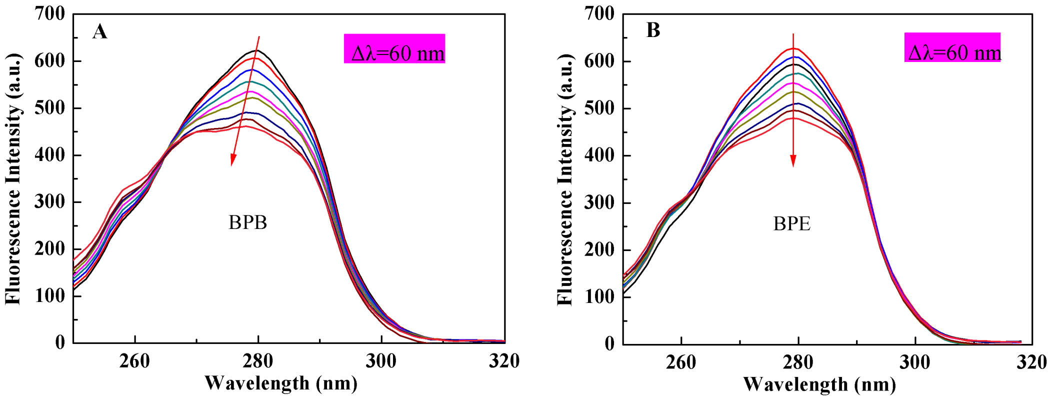

Supplementary Figure 1. Synchronous fluorescence spectra of HSA in the presence of different concentrations of BPB **(A)** and BPE **(B)** at Δλ = 60 nm. Concentrations of BPB/BPE were in the range of 0–120 μM.
